# Antineoplastic Activity and Curative Role of Avenanthramides against the Growth of Ehrlich Solid Tumors in Mice

**DOI:** 10.1155/2019/5162687

**Published:** 2019-01-13

**Authors:** Maha A. Aldubayan, Rehab M. Elgharabawy, Amira S. Ahmed, Ehab Tousson

**Affiliations:** ^1^Department of Pharmacology & Toxicology, Faculty of Pharmacy, Qassim University, Saudi Arabia; ^2^Department of Pharmacology & Toxicology, Faculty of Pharmacy, Tanta University, Tanta, Egypt; ^3^Department of Hormones, Medical Research division, National Research Centre, Cairo, Egypt; ^4^Department of Zoology, Faculty of Science, Tanta University, Tanta, Egypt

## Abstract

Interest is growing in finding natural sources of effective antitumor agents that generate fewer side effects than conventional chemotherapeutic drugs. Avenanthramides (Avns) are such compounds; these phenolic molecules naturally occur in oats and have antioxidant, anti-inflammatory, and antiproliferative effects making them worthy of further research. The aim of this study is to characterise Avns' curative ability and antineoplastic activity on solid-form Ehrlich tumors. For the study, 75 female mice were randomly and equally allocated to five groups (group 1-control, group 2-DMSO, group 3-positive control receiving Avns, group 4-mice with Ehrlich solid tumor, and group 5-Ehrlich solid tumor treated with Avns). Mice with Ehrlich solid tumors exhibit increased tumor volume; elevated expression of AFP, ALT, AST, Bcl2, CEA, cholesterol, creatinine, urea, MDA, PCNA, potassium, triglycerides, TNF-*α*, and NF-*κ*B; and a concomitant decline in catalase, GSH, P53, and SOD. In the mice with Ehrlich tumors who received Avns, there appeared to be improvement in NF-*κ*B TNF-*α*, tumor markers (AFP and CEA), electrolytes, liver and kidney function enzymes, and lipid profiles; reduced MDA level; improved antioxidant parameters; normalised liver protein, P53, and PCNA; and reduced Bcl2 expression. Pathological examination of tumor lesions also indicated improvement. These results suggest that Avns exhibit antineoplastic activity and possess antioxidant properties that enhance the antioxidant defence system, thus reducing the oxidative stress caused by Ehrlich solid tumors.

## 1. Introduction

Alongside cardiac disease, infectious disease, malnutrition, and war, cancer is one of the foremost causes of mortality worldwide. Cancer is the loss of the normal cell cycle, resulting in the unregulated cell growth and the lack of differentiation, characterised as malignant growths. Cancer can occur at any time, in any tissue or organ [1, 2]. The Ehrlich tumor is a spontaneous murine mammary adenocarcinoma. It is an aggressive carcinoma that can occur in almost all strains of mice [3]. The solid form of the tumor is undifferentiated, making particularly useful for studies of tumors. Not only is this type of tumor used to develop tumor models but also it is used in chemotherapy studies [4]. As an alternative to conventional anticancer treatments, novel drugs are being created from the secondary metabolites of plants; these compounds often appear to be less toxic and/or more effective than traditional chemotherapy treatments [5, 6]. These attributes have stimulated interest in identifying antitumor agents derived from natural sources.

According to Kim et al. [7], the plant kingdom may be a significant source of numerous compounds that have antitumor and cytotoxic capabilities. It is already recognised that because of the fibre content, a whole grain-rich diet promotes health; however, whole grain cereals, such as oats, are also significant sources of health-benefitting, phenolic compounds. Avenanthramides (Avns; anthranilic acid amides) are a group of phenolic alkaloids found mainly in oats (*Avena sativa*), but also present in white cabbage. Avns are one such phenolic compound identified in oats, which, due to their antioxidant, anti-inflammatory, and antiproliferative effects, are considered beneficial to health [8, 9]. These properties identify oats as a valuable food, capable of protecting against coronary heart disease, diabetes, and cancer [10, 11]. AVAs are polyphenols belonging to a group of hydroxycinnamoylanthranilate alkaloids; these low-molecular-weight compounds are comprised of amide conjugates of anthranilic acid or its hydroxylated derivatives, and hydroxycinnamic or avenalumic acids [12].

Avenanthramides have an anti-inflammatory effect through reduction in the activity of nuclear factor-kappa *β* (NF-*κ*B) in NF-*κ*B-dependent cytokine [13]. Regulation of the transcription of DNA and the activation of genes related to inflammatory and immune responses are the responsibility of NF-*κ*B [14]. Inhibition of NF-*κ*B reduces cell proliferation and inflammation [11]. Avenanthramides are able to inhibit the release of inflammatory cytokines that are present in pruritic skin diseases and may cause the itch sensations. Therefore, the current study is aimed at describing the antineoplasic activity and the curative role of avenanthramides (Avns) on Ehrlich tumor in the solid forms.

## 2. Materials and Methods

### 2.1. Chemicals

#### 2.1.1. Avenanthramides

Avenanthramide-C methyl ester (CAS number: 955382-52-2; catalogue number: CAY10011336-1 mg) was obtained from Cayman Chemical (Ann Arbor, MI 48108, USA). Avenanthramides were solved in DMSO.

### 2.2. Animals

In total, 75 Albino, female mice individually weighing around 20–25 g were housed in wire mesh cages in a constant environment [temperature (23°C ± 2°C), relative humidity (80% ± 5%), and light (12 h light/dark cycles)]. They were given ad lib access to a standard diet and water. All experiments were performed in accordance with guidelines for animal studies issued by the Ethical Committee of Faculty of Science, Tanta University, as approved by the Institutional Animal Care and Use Committee (IACUC-SCI-TU-0041).

### 2.3. Ehrlich Ascites Carcinoma (EAC) Cells and Tumor Inoculation

Mice with Ehrlich ascites carcinoma (EAC) were purchased from the Egyptian National Cancer Institute (NCI; Cairo University, Egypt). The female Swiss albino mice were intraperitoneally injected with 2.5 million cells per mouse to maintain the tumor line. Prior to intraperitoneal injection, the EAC cells were counted using a bright line haemocytometer; dilution was performed using a physiological sterile saline solution. A volume of 0.2 mL containing the desired number of cells was injected. To assess Ehrlich solid tumors (Ehrlich), 0.2 mL EAC cells (2.5 × I0^6^ mouse cells) were subcutaneously inoculated into the left thigh of the mouse's rear limb.

### 2.4. Animal Treatments

The mice were given one week to acclimatise, then they were randomly divided into five equal groups (15 mice per group).

Group 1 (G1): negative control; as negative controls, these mice were untreated
Group 2 (G2): DMSO: 50 mg/kg of DMSO were administered by oral gavage, 3 times per week, for two weeks [15]
Group 3 (G3): negative control receiving Avns; doses of 50 ng/kg/day were administered by oral gavage for two weeks [14]
Group 4 (G4): mice with Ehrlich solid tumor [16]
Group 5 (G5): cotreatment of Avns and Ehrlich solid tumor: for two weeks, starting at day zero of the Ehrlich induction, mice were treated with Avns

### 2.5. Blood Sampling

After completion of the procedure, animals were euthanized with intraperitoneal injection with sodium pentobarbital and subjected to a complete necropsy. Blood samples were obtained from the inferior vena cava of each rat and collected in nonheparinised glass tubes and left for 30 min at room temperature to clot. The samples were then centrifuged at 5000 rpm for 10 min. Sera were separated and stored in aliquots at −80°C until required. Prior to assay, samples were thawed at room temperature.

#### 2.5.1. Measuring Alpha Fetoprotein Tumor Marker

An automated quantitative enzyme-linked fluorescent assay (ELFA) using mini-VIDAS® AFP (bioMérieux, Marcy-l'Etoile, France) was used to measure alpha-fetoprotein (AFP).

#### 2.5.2. Determining Carcinoembryonic Antigen Tumor Marker

Carcinoembryonic antigen (CEA) was measured by using the quantitative sandwich immunoassay, MyBioSource Mouse Carcinoembryonic Antigen Elisa Kit (MyBioSource, San Diego, USA).

#### 2.5.3. Determining the Level of Tumor Necrosis Factor Alpha (TNF-*α*)

A quantitative sandwich enzyme immunoassay (R&D systems, Minneapolis, USA) was performed using TNF-*α*-specific monoclonal antibodies in accordance with the manufacturer's instructions.

#### 2.5.4. Serum Liver Function Enzymes

Both aspartate transaminase (AST) and alanine transaminase (ALT) activities in serum were assayed by using a commercial kit that was supplied by HumanN (Germany) according to the method of Reitman and Frankel [17].

#### 2.5.5. Serum Kidney Function Enzymes

The level of serum urea was analyzed by using a commercial kit that was purchased from Diamond, Egypt. Serum urea was determined according to the method of Patton and Crouch [18]. Serum creatinine concentration was measured by using a commercial kit that was supplied by Diamond, Egypt. Serum creatinine was determined according to the method of Larsen [19]. Serum sodium and potassium concentration was assayed by a colorimetric method using a commercial kit supplied by Diatek Company [20].

#### 2.5.6. Serum Lipid Profile

Commercial assay kits, Spinreact (Santa Coloma, Spain), were used to measure the serum level of cholesterol following the method proposed by Allain et al. [21], whereas triglyceride (TG) levels were established using the method of Mesbah et al. [22].

### 2.6. Tissue Sampling

Tumors were carefully removed from the carcasses, weighed, and their dimensions measured. To remove extraneous materials, tumor tissues were then washed with ice-cold saline three times then chilled on ice. Tumors were divided into four sections, wrapped in aluminium foil, and stored at −80°C until required for the preparation of tissue homogenates [23]. The same process was performed for isolated Ehrlich tumors from the right thigh. Excised tumors were weighed in grams and the volume of the tumor mass that had developed measured [24].

#### 2.6.1. Preparing the Tissue

After the two-week experiment, the mice were fasted, anaesthetised, and euthanized with ether; then, 10-12 hours after fasting, a full necropsy was performed. Tumor tissues were homogenised using ice-cold 1.15% KCl and 0.01 mol/L sodium potassium phosphate buffer (pH 7.4) and a Potter-Elvehjem-type homogeniser to achieve 10% *w*/*v* homogenisation. The homogenate was centrifuged at 4°C for 20 min at 10,000 g. The resultant supernatant was used for enzyme assays.

#### 2.6.2. Determining Ehrlich Tumor-Oxidative Stress Biomarkers

To determine the malondialdehyde (MDA) component, a noxious product of lipid peroxidation was detected according to the following: lipid peroxidation was evaluated on the basis of MDA level, and MDA in liver was determined using the method described by Mesbah et al. [25]. Assay of catalase enzyme activity (CAT; EC 1.11.1.6) was measured by monitoring the decomposition of H_2_O_2_ (the substrate of the enzyme) at 240 nm according to the method described by Aebi [26]. Assay of superoxide dismutase enzyme activity (SOD; EC 1.15.1.1) in liver homogenate was assayed by the method of Habig et al. [27]. Assay of reduced glutathione (GSH) content determination is based on the reduction of 5,5-dithiobis(2-nitrobenzoic acid), and it was determined by the colorimetric method according to Ellman [28].

### 2.7. Histopathological Studies

Immediately after dissection, Ehrlich tumors were removed and fixed in 10% neutral buffered formalin. Fresh isolated tumor from different groups was stained using routine haematoxylin and eosin counterstain methods, in accordance with Bancroft and Cook [29].

### 2.8. Immunohistochemical Detection of Bc1-2, p53, PCNA, and NF-*κ*B Expression

The avidin-biotin complex (ABC) method was used to assess the immunoreactivities of p53, Bc1-2, PCNA, and NF-*κ*B [14, 30]. Paraffinised sections (5 *μ*m thick) of fixed tumor that had been mounted on gelatin chrom alum–coated glass slides were dewaxed. To reduce nonspecific background staining, sections were then washed for 5 min with distilled water, followed by a PBST rinse for 10 min before being incubated with 10% normal goat serum for 15 min. Then, the sections were incubated with anti-rabbit p53 or anti-rabbit Bc1-2 or anti-rabbit PCNA (monoclonal antibody, Dako; 1 : 80, 1 : 2000, and 1 : 100, respectively) or rabbit polyclonal anti-NF-*κ*B/p65 (Rel A) Ab-1 antibody (Cat. No. RB-1638-R7, Ready-to-Use, Lab Vision) at room temperature for 1–2 h. The manifestation of a dark, brown-coloured, intracytoplasmic precipitate is indicative of p53, Bcl-2, PCNA, and NF-*κ*B being present. The primary antibody was excluded in the negative control; this was to protect against any false-positive results that could arise nonspecific reactions.

### 2.9. Statistical Analysis

The analysis of results was done using the Statistical Package for the Social Sciences (SPSS software version 16). Data were presented as mean ± standard error of mean (SEM) and statistically analyzed by one-way ANOVA (analysis of variance) followed by Dunnett test. Dunnett test comparisons were performed to assess the significance of differences between groups. Unpaired *T*-test was performed to compare the significant difference between groups. The criterion for statistical significance was set at *P* < 0.05.

## 3. Results

### 3.1. Effect of Avns on Ehrlich Tumor Volume

**Figure 1:**
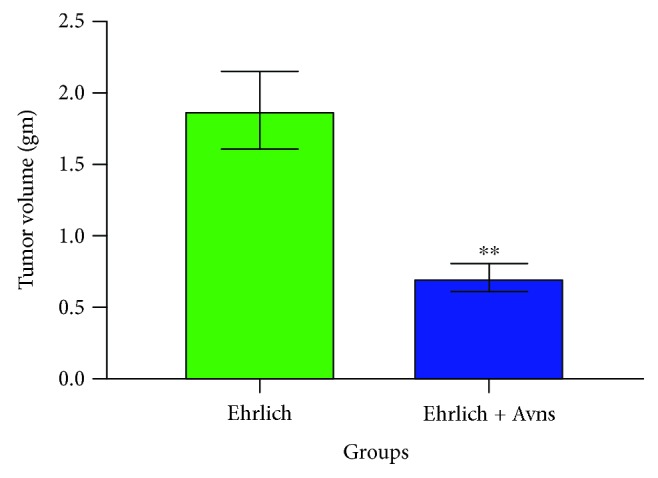
Effect of avenanthramides (Avns) on mouse Ehrlich solid tumor volume. ^∗∗^*P* value = 0.0037 (two-tailed). The significant difference was analyzed by unpaired *T*-test. Values are expressed as mean ± SEM. The *T*-test was significant at *P* < 0.05. The unpaired *T*-test was significant from corresponding Ehrlich at ^NS^*P* = 0.1234, ^∗^*P* = 0.0332, ^∗∗^*P* = 0.0021, ^∗∗∗^*P* = 0.0002, and ^∗∗∗∗^*P* < 0.0001.

The effect of Avns treatment upon the growth and proliferation of subcutaneously injected Ehrlich cells is presented in Figure 1. After 14 days of administering Ehrlich injections, growth-dependent changes were determined by assessing the volume of tumors in the different groups. The data show that compared with G4, there was a significant decrease (^∗∗^*P* = 0.0037) in tumor volume in G5 mice.

### 3.2. Changes in Tumor Markers

**Figure 2:**
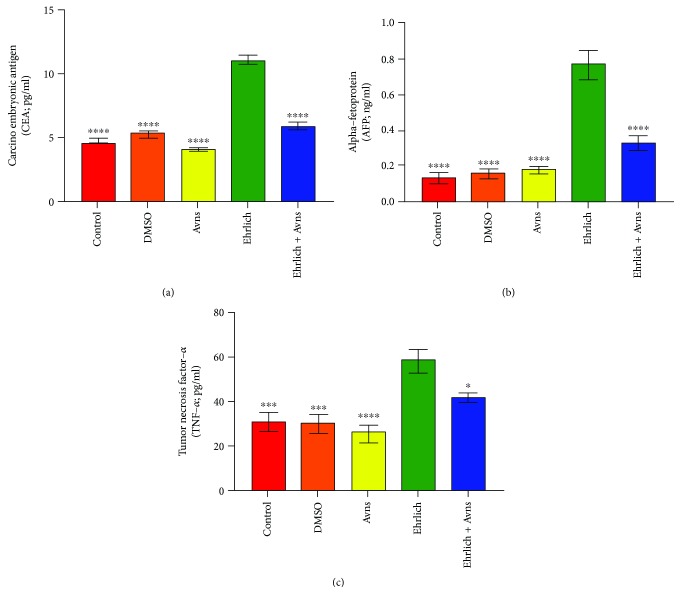
Alternation in the carcinoembryonic antigen (CEA), alpha-fetoprotein (AFP), and tumor necrosis factor alpha (TNF-*α*) levels in different experimental groups. The significance of difference was analyzed by one-way ANOVA and Dunnett test (compare all vs. Ehrlich group). Values are expressed as means ± SEM. One-way ANOVA was significant at *P* < 0.05. Dunnett test was significant from the corresponding Ehrlich group value at ^∗^*P* < 0.05, ^∗∗^*P* < 0.01, ^∗∗∗^*P* < 0.001, and ^∗∗∗∗^*P* < 0.0001.

Figure 2 shows a significant increase in serum alpha fetoprotein (AFP) and plasma carcinoembryonic antigen (CEA) levels in the Ehrlich solid tumor group (Ehrlich) when compared with the control group. On the other hand, there was a significant decrease in AFP (*P* = 0.0001) and CEA (*P* = 0.0001) levels in the cotreated Ehrlich solid tumor group with avenanthramides (Avns) groups when compared with the Ehrlich group (*P* < 0.0001) (Figure 2).

### 3.3. Effect of Avns on Plasma on Levels of TNF-*α*

Figure 2 depicts a significant increase (*P* = 0.0001) in the plasma levels of TNF-*α* in G4 compared to the control groups (*P* = 0.0005). Also, the levels of TNF-*α* in G5 (*P* = 0.0342) were significantly lower than the levels detected in G4.

### 3.4. Effect of Avns on Liver Enzymes

Comparing the serum levels of ALT and AST in G4 with the other groups shows that in G4 (*P* < 0.0001), the enzyme levels were significantly greater than G1 (*P* = 0.0001), G3 (*P* value = 0.0001), and G5 (*P* = 0.0001) (Table 1).

### 3.5. Effect of Avns on Kidney Function and Electrolyte Levels

Compared to G1 and G3, G4 showed a significant increase in serum levels of creatinine (*P* < 0.0001), urea (*P* < 0.0001), and potassium (*P* = 0.0286) (Table 1). G4 also showed a significant decrease (*P* = 0.0080) in the serum level of sodium compared to the control group (*P* = 0.0207) (Table 1).

### 3.6. Effect of Avns on Lipid Profile Levels

As Table 1 shows, the levels of serum cholesterol and triglycerides were significantly increased in G4 compared to G1 and G3. The biochemical lipid profile of G5 was improved, as indicated by a significant reduction in the levels of cholesterol (*P* value = 0.0002) and triglycerides (*P* = 0.0010) compared to the levels observed in G4 (*P* < 0.0001) (Table 1).

### 3.7. Effects of Avns upon Markers of Oxidative Stress

**Figure 3:**
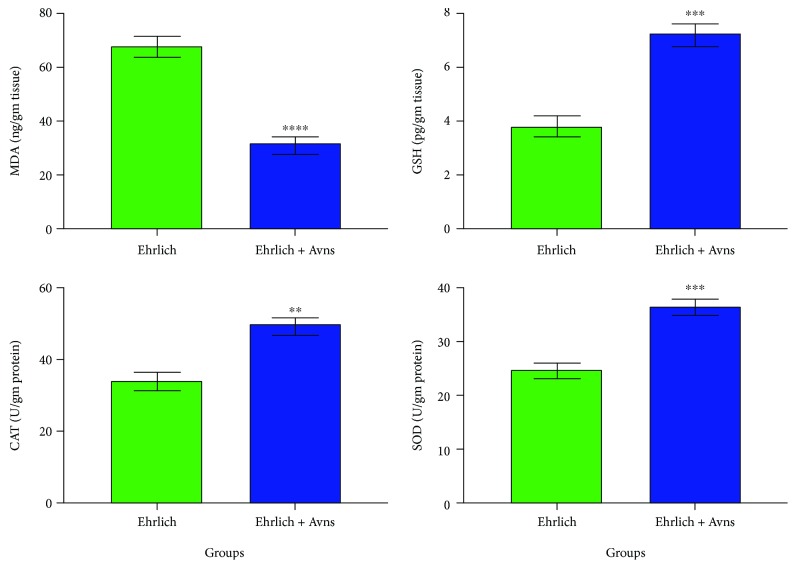
Changes in malondialdehyde (MDA) (^∗∗∗^*P* value < 0.0001; two-tailed), reduced glutathione (GSH) (^∗∗∗^*P* value = 0.0002; two-tailed), catalase (CAT) (^∗∗^*P* value = 0.0019; two-tailed), and superoxide dismutase (SOD) (^∗∗∗^*P* value = 0.0005; two-tailed), activities in tumors homogenate in Ehrlich and posttreated Ehrlich with Avns groups. The significant difference was analyzed by unpaired *T*-test. Values are expressed as mean ± SEM. The *T*-test was significant at *P* < 0.05. The unpaired *T*-test was significant from corresponding Ehrlich at ^NS^*P* = 0.1234, ^∗^*P* = 0.0332, ^∗∗^*P* = 0.0021, ^∗∗∗^*P* = 0.0002, and ^∗∗∗∗^*P* < 0.0001.

Figure 3 reveals that Ehrlich solid tumor- (Ehrlich-) induced toxicity; depletion in the levels of CAT, GSH, and SOD; and an increase in the level of MDA in tumor tissue were detected. On the other hand, treatment of the Ehrlich solid tumor group with Avns revealed a significant (*P* < 0.05) increase in the levels of CAT, GSH, and SOD and a significant (*P* < 0.05) decrease in the level of MDA in tumor tissue as compared with the Ehrlich solid tumor group (Figure 3).

### 3.8. Effect of Avns on the Histopathology of Ehrlich Solid Tumors

**Figure 4:**
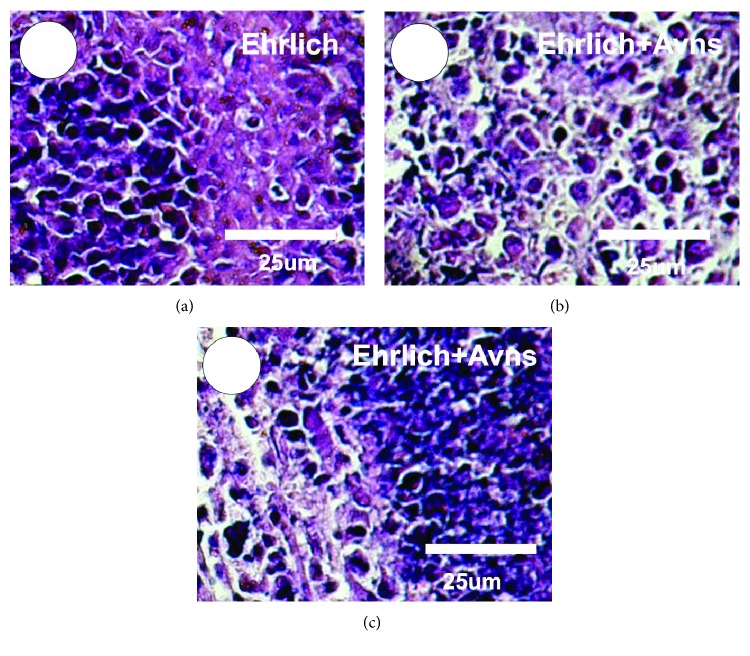
Photomicrographs represent subcutaneous Ehrlich solid tumor in mice stained with haematoxylin & eosin. (a) Ehrlich solid tumor with groups of large, round, and polygonal cells, with pleomorphic shapes, hyperchromatic nuclei, and binucleation. (b, c) Treated Ehrlich solid tumor with Avns showed a high regression of tumor development, spread within the muscle tissue, wide zones of apoptotic cells, and many zones of tumor cell remnants.

Histopathological examination of the Ehrlich solid tumor (Ehrlich) under a light microscope showed compact aggregation of the tumor tissue cells which spread within the muscular tissues (Figures 4(a) and 4(b)). Subcutaneously implanting Ehrlich tumour cells led to development of solid tumours; these were manifest as sheets of small, highly chromatophilic tumour cells with inconsistent morphology. This represents cell proliferation and proximal regions of necrosis and differentiated cells (Figure 4(b)). Ehrlich solid tumor showed groups of large, round, and polygonal cells, with pleomorphic shapes, hyperchromatic nuclei, and binucleation. In addition, several degrees of cellular and nuclear pleomorphisms were also observed (Figure 4(b)). Cotreatment of Ehrlich solid tumor with Avns revealed a high regression of tumor development, high and wide zones of apoptotic cells, and other many zones of tumor cells remnants observed (Figure 4(c)).

### 3.9. Detection of Antiapoptotic Bcl2 Protein Expression in Ehrlich

**Figure 5:**
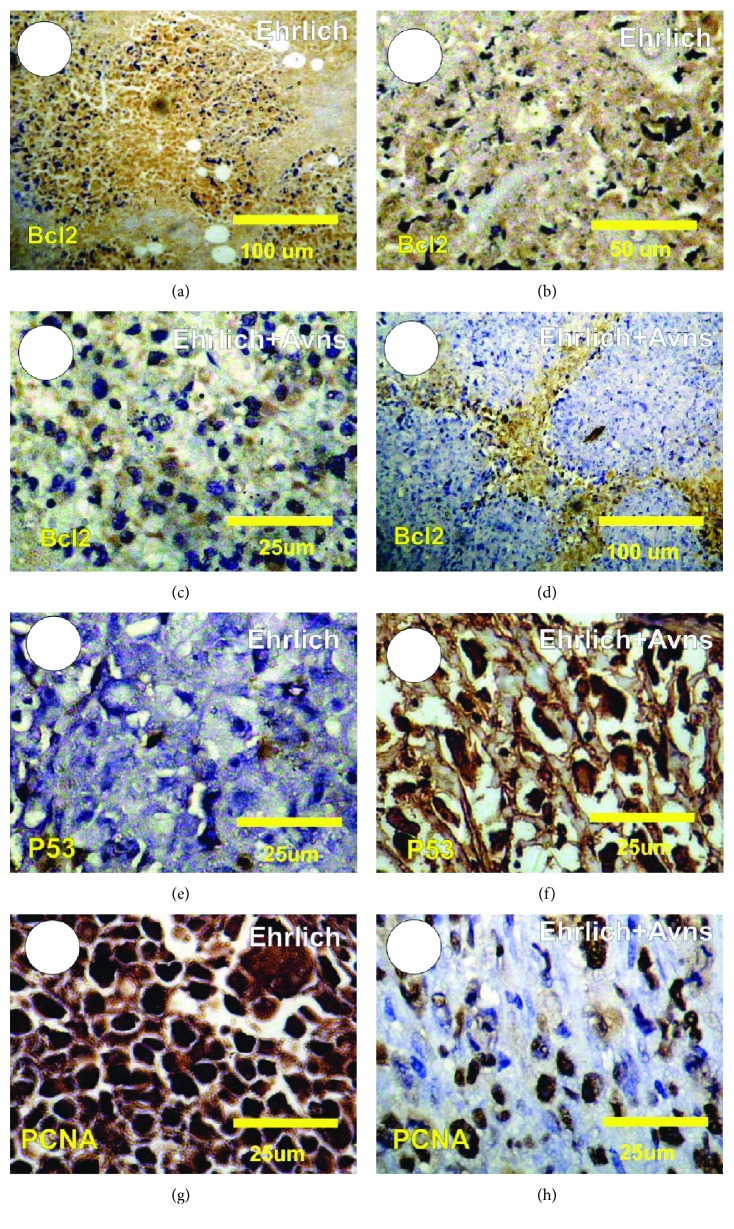
Photomicrograph of tumor sections in control and treated Ehrlich solid tumor with Avns stained with antiapoptotic Bcl2, apoptotic P53, and proliferating PCNA markers. (a, b) Tumor sections in Ehrlich solid tumor revealed strong positive reactions for Bcl2 (brown color). (c, d) Tumor sections in treated Ehrlich solid tumor with Avns revealed mild to moderate positive reactions for Bcl2 (brown color). (e) Tumor sections in Ehrlich solid tumor revealed faint positive reactions for P53. (f) Tumor sections in treated Ehrlich solid tumor with Avns revealed strong positive reactions for P53 (brown color). (g) Tumor sections in Ehrlich solid tumor revealed strong positive reactions for PCNA. (h) Tumor sections in treated Ehrlich solid tumor with Avns revealed mild to moderate positive reactions for PCNA (brown color).

The detection of Bcl2 expression in tumor sections in the Ehrlich solid tumor (Ehrlich) and Ehrlich cotreated with Avns is revealed in Figures 5(a)–5(d). The tumor section in the Ehrlich group shows a strong positive reaction for Bcl2 expression, while mild to moderate positive reactions for Bcl2 expression were detected in Ehrlich cotreated with Avns (Figures 5(a)–5(d)).

### 3.10. Detection of Apoptotic P53 Protein Expression in Ehrlich

The detection of P53 expression in tumor sections in the Ehrlich solid tumor (Ehrlich) and Ehrlich cotreated with Avns is revealed in Figures 5(e) and 5(f). The tumor section in the EST group shows a faint positive reaction for P53 expression, with strong positive reactions for P53 expression detected in Ehrlich cotreated with Avns (Figures 5(e) and 5(f)).

### 3.11. Detection of PCNA Expression in Ehrlich

The detection of PCNA expression in tumor sections in Ehrlich solid tumor (Ehrlich) and Ehrlich cotreated with Avns is revealed in Figures 5(g) and 5(h). The tumor section in the Ehrlich group shows a moderate positive reaction for PCNA expression, while mild to moderate positive reactions for PCNA expression were detected in Ehrlich cotreated with Avns (Figures 5(g) and 5(h)).

### 3.12. Detection of Nuclear Factor-Kappa *β* (NF-*κ*B) Expression in Ehrlich

**Figure 6:**
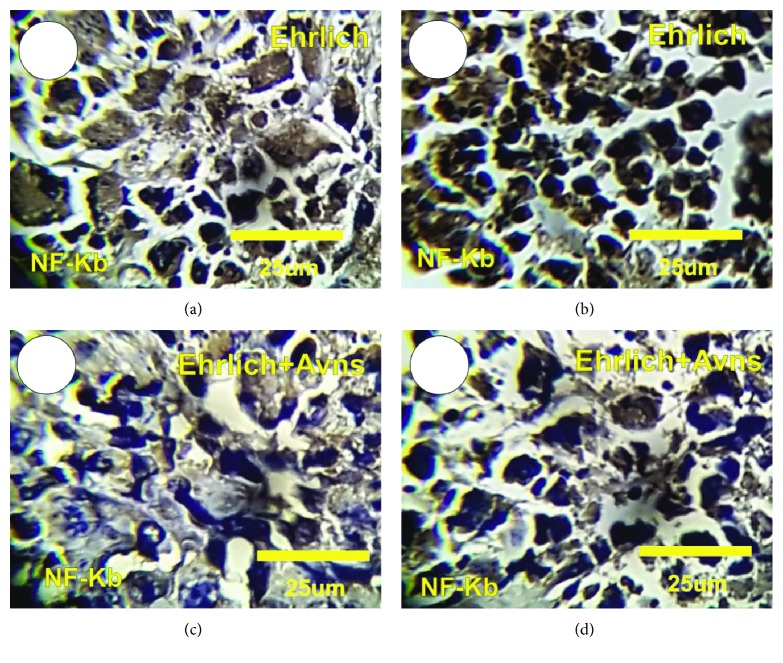
Photomicrograph of tumor sections in control and treated Ehrlich solid tumor with Avns stained with nuclear factor-kappa *β* (NF-*κ*B) markers. (a, b) Tumor sections in Ehrlich solid tumor revealed strong positive reactions for NF-*κ*B (brown color). (c, d) Tumor sections in treated Ehrlich solid tumor with Avns revealed mild positive reactions for NF-*κ*B (brown color).

The detection of NF-*κ*B expression in tumor sections in the Ehrlich solid tumor (Ehrlich) and Ehrlich cotreated with Avns is revealed in Figures 6(a)–6(d). The tumor section in the Ehrlich group shows a moderate to strong positive reaction for NF-*κ*B expression, while the tumor section in Ehrlich cotreated with Avns inhibits NF-*κ*B, where mild NF-*κ*B-positive reactions for NF-*κ*B expression were detected in Ehrlich cotreated with Avns (Figures 6(a)–6(d)). The intensity of NF-*κ*B expressions was significantly decreased in Ehrlich + Avns as compared with Ehrlich tumor sections.

## 4. Discussion

Avenanthramides (Avns), unique polyphenols found exclusively in oats and oatmeal, exhibit antioxidant and anti-inflammatory activity [8, 14]. In this regard, the present study was undertaken to evaluate the potential protective and curative effect of Avns on Ehrlich solid tumor cell-inoculated mouse-induced carcinogenesis. The current study was done on Ehrlich solid tumor because of its simple ability for induction in mice; also, this type of Ehrlich tumor which appeared originally as a spontaneous breast carcinoma in a mouse can resemble the breast cancer [31].

Control or untreated mice that were intramuscularly inoculated with Ehrlich ascites developed a solid tumor in the right thigh of the hind limb within 13 days of inoculation. This is consistent with previous research based on the same model [32]. Following cotreatment of Ehrlich solid tumor with Avns, there was a distinct reduction in the size of the tumor size; moreover, the tumors appeared to grow slowly and their morphology was discontinuous and fragmented. This indicates that Avns exerted some inhibitory effects upon the tumor cells. The results of the study show that Avns demonstrates antitumor activity against Ehrlich solid tumor, as evidences by reduced tumor weight and volume (data not displayed). These findings are in accordance with those of Guo et al. [14] who noted that Avns inhibited proliferation of human colon cancer cell lines.

Cancer patients often suffer from the condition, cachexia, in which there is a progressive loss of fat and muscle mass leading to overwhelming weakness; it is attributed to the action of cytokines such as TNF-*α* and IFN-*γ*, which are released by macrophages [9, 33]. TNF is a highly pleiotropic cytokine that has a key role in host defence, immune homeostasis, and inflammation [34]. In this study, the plasma levels of TNF-*α* were significantly elevated in G4 compared to G1 and G3. This increase in TNF-*α* levels in the tumor-afflicted mice could be due to a rise in macrophage-produced ROS, which increases lipid peroxidation. This is considered to be important by pathogenically contributing to liver damage [35]. The results of the study are consistent with those of Abd El-Dayem et al. [36] who reported elevated TNF-*α* levels in female mice with Ehrlich ascites carcinoma. However, the results are inconsistent with those generated by Mansour and Anis [16] who noted that the plasma concentrations of TNF-*α* and IL-10 in Ehrlich solid tumor-bearing mice were significantly decreased compared to controls. Cotreatment of Ehrlich solid tumor with Avns depletes the increase in TNF-*α* levels in Ehrlich solid tumor. As postulated by Guo et al. [14], the mechanism responsible for this may be that Avns has excellent ability in scavenging diverse free radicals.

Serum AFP is one of several tumor markers that become elevated where certain cancers are present. In the G4 group of this study, the levels of serum AFP and CEA were found to be significantly greater than in G1, G3, and G5. Based on these findings, the indications are that in G4, liver and kidney functions were increased. This upregulation might be indicative of hepatic and renal damage caused by the effect of cancer cell invasions.

Liver damage induced by tumor cells typically indicates disruption of normal liver cell metabolism, resulting in changes in the activity of serum enzymes. In the current study, there was a significant increase in the serum levels of ALP, ALT, and AST, together with a concomitant decline in the levels of serum albumen and total proteins in G4 mice, indicating liver toxicity. The increase in liver enzymes might arise from a generalised destruction of hepatocytes and the release of AST into the plasma following the induction of the tumor. Albumin, which is the most abundant plasma protein, and other plasma proteins are synthesised in hepatocytes in the liver; they are useful, easily monitored markers of liver function [37]. Thus, the significant decline in the level of serum albumin and total proteins detected in the mice with Ehrlich solid tumors corresponds to liver damage, which manifests as reduced biosynthetic capabilities. To reiterate, the damage caused by Ehrlich solid tumors may reduce the liver's ability to synthesise plasma proteins. These results echo those of Gupta et al. [38] who reported that liver transaminases were increased in mice with Ehrlich ascites cancer cells, suggesting liver dysfunction. Comparable results were obtained by Abou-Zaid et al. [39]. A number of studies have noted elevated levels of ALT and AST attributed to hepatotoxicity stimulated by diverse carcinogens in various animal models [40, 41]. The recorded rise in serum levels of ALP, ALT, and AST might be interpreted as a consequence of liver damage or as changes to the permeability of membranes, which suggests that Ehrlich solid tumors cause severe hepatocellular damage.

Also, in the present study, treatment with Avns caused significant attenuation of this elevation in ALT, AST, and ALP liver enzymes and came back to be close to normal values indicating the antitumor effect of Avns agent. Equally, the level of serum albumin, which had become considerably depressed in G4 mice, was improved in G5 mice that all received treatment of Avns. Elevated serum levels of creatinine and urea are indicative of renal dysfunction. In the present study and consistent with the results observed by Abou-Zaid et al. [39], Hussein and Azab [42], and Hussein [43], there was a significant increase in the serum levels of creatinine and urea in G4 mice. The increase in the concentration of blood urea is ascribed to the catabolic effect of tumors and the subsequent increase in the production of urea. Also, there was a significant increase in the concentration of serum potassium and a decrease in the concentration of sodium in G4 mice, compared to G1 and G3 mice. This indicates changes in the balance of electrolytes and renal toxicity. The hepatic and renal toxicity associated with tumorigenesis may arise from oxidative damage caused by the excessive production of ROS [44] and cytokines (TNF-*α*) [45, 46].

Oxidative stress is considered key in the development of numerous health conditions including cardiovascular diseases and obesity. The evidence also indicates it has a major role in tumorigenesis [47, 48]. Studies of diverse cereal components find that the antioxidant activity of (Avns) is 10–30 times greater than ferulic acid, gentisic acid, p-hydroxybenzoic acid, protocatechuic acid, syringic acid, vanillic acid, and vanillin [49, 50]. These radicals can encourage tumor growth and metastasis by promoting the tumor cells' invasive, angiogenic, and migratory abilities [50, 51].

This study found a positive correlation between the changes in antioxidant mechanisms, apoptosis, and the proliferation of Ehrlich solid tumor cells. There was a statistically significant increase in the level of MDA and a decrease in the levels of catalase, GSH, and SOD in the tissues received from the G4 tumors. As stated earlier in this report, Avns have the ability to modulate antioxidant enzyme activity by promoting the levels of catalase, GSH, and SOD, as well as inhibiting the level of MDA. Marklund and Marklund [52] also reported that tumor growth inhibited catalase and SOD activity. These results agree with other studies which detected elevated levels of MDA in breast cancer [53]. The findings here are consistent with those of Abd El-Aziz et al. [51] who noted that Ehrlich tumors exhibited significant increases in MDA and considerable decreases in catalase and SOD catalase. Moselhi and Al Mslmani [54] propose that the diminished levels of SOD levels may arise from the altered antioxidant status initiated by carcinogenesis. Macromolecules such as lipids become damaged through oxidative stress brought on by the excessive production of free radicals; *in vivo*, this can induce lipid peroxidation [38, 55]. The reduced levels of GSH found in tumor-bearing mice might be due to the transformation rate of GSH to oxidised GSH increasing in an effort to reduce the intracellular concentration of hydrogen peroxide. As the aforementioned results indicate, treating the tumor with Avns promotes liver and kidney function, to improve the levels of tumor marker compounds, such as TNF-*α*, and increase antioxidant parameters to reduce oxidative stress.

Carcinogenesis is effectively the loss of equilibrium between the process of cellular proliferation and apoptosis. Elevated rates of apoptosis have been linked with inhibiting the growth of cells that are potentially oncogenic [56]. The apoptosis pathway is important and one that antitumor drugs are aimed at exploiting [57]. The influence of programmed cell death is important to tumor progression and is regarded as the primary mechanism responsible for tumor cell death following chemotherapy, radiation therapy, and immunotherapy interventions [44]. The results presented here indicate that in the tumor tissues from G4 mice there was a positive expression of Bcl2, PCNA, and NF-*κ*B and a negative expression of P53. Evaluations of cell proliferation are one of the key indicators used to determine cancer prognoses. In clinical settings, the number of cells undergoing mitosis is counted to assist with the histopathological grading or labelling of cells in the S phase. PCNA is also an important parameter of proliferation, which has an important role in initiating the onset of the cell cycle and increasing the G1-S phases of the cell cycle [58]. In this study, PCNA expression in the tumors retrieved from G5 mice was reduced, which is attributed to the administration of Avns.

The regulation of numerous cellular processes such as proliferation, apoptosis, and survival is done through the nuclear factor kappa B (NF-*κ*B) pathway, which has emerged as an important therapeutic target in cancer. Activation of the NF-*κ*B transcription factor is associated with nuclear translocation of the p65 component of the complex. NF-*κ*B regulates the expression of various molecules important in tumorigenesis, such as matrix metalloproteinases, cyclooxygenase-2 (COX-2), iNOS, chemokines, and inflammatory cytokines, all of which promote tumor cell invasion and angiogenesis [59]. Our immunohistochemical results indicate that the expression of NF-*κ*B was inhibited in tumor sections treated with Avns through the inhibition of NF-*κ*B-dependent intracellular signalling.

According to Glantz et al. [60], there is evidence to indicate that defective apoptosis could arise from the abnormal expression of Bcl-2 and enhanced expression of caspase-3. When exposed to exogenous damaging stimuli, the cells activate the regulators of expression for these genes. The P53 tumor-suppressor protein is one such transcription factor that controls the rate of transcription of a number of genes implicated in cell cycle regulation, DNA repair, and apoptosis [61]. Therefore, it is important in protecting the cell against apoptosis without affecting cell proliferation. Cells become more resistant to apoptosis should there be a surfeit of antiapoptotic proteins [62]. The results of this study reveal that there was an inverse correlation between the expression of Bcl-2 and p53 proteins. Tousson et al. [30] generated comparable results, observing an inverse relationship between the patterns of expression of Bcl-2 and p53 proteins. Thus, in the present study, the increase in expression of proapoptotic p53 and the decrease in antiapoptotic Bcl-2 antiapoptotic suggest a rise from the administration of Avns.

## 5. Conclusions

Avenanthramides (Avns) are important phenolic compounds found in oats, which have beneficial health properties because of their antioxidant, anti-inflammatory, and antiproliferative effects. Here, we describe the antineoplasic activity and the curative role of avenanthramides (Avns) on Ehrlich tumor in the solid forms. Avenanthramides revealed potent antineoplasic activity and antioxidant properties by augmenting the antioxidant defence system hereby protecting the tumor against oxidative stress induced by Ehrlich solid carcinoma tumors.

## Figures and Tables

**Table 1:** Effects of avenanthramides (Avns) on biochemical parameters in serum of Ehrlich solid tumor- (EST-) bearing mice serum.

	Control	DMSO	Avns	Ehrlich	Ehrlich + Avns
ALT (U/L)	29.6±1.56^∗∗∗∗^	33.1±2.48^∗∗∗∗^	28.0±2.12^∗∗∗∗^	74.6 ± 3.05	36.5±2.93^∗∗∗∗^
AST (U/L)	59.8±4.51^∗∗∗∗^	63.0±3.45^∗∗∗∗^	58.7±3.88^∗∗∗∗^	112.8 ± 7.15	71.5±6.72^∗∗∗∗^
Creatinine (mg/dL)	0.41±0.09^∗∗∗∗^	0.46±0.04^∗∗∗∗^	0.37±0.11^∗∗∗∗^	1.16 ± 0.06	0.75±0.17^∗∗^
Urea (mg/dL)	26.53±0.96^∗∗∗∗^	27.04±1.74^∗∗∗∗^	24.36±1.33^∗∗∗∗^	40.01 ± 2.25	31.70±1.89^∗∗∗∗^
Na ions (mEq/L)	134.5 ± 9.8^∗^	138.4 ± 8.5^∗^	144.9±9.3^∗∗^	104.5 ± 8.4	131.8 ± 9.6^∗^
K ions (mEq/L)	4.15 ± 0.35^∗^	4.45 ± 0.18^∗^	4.21 ± 0.35^∗^	5.33 ± 0.46	4.38 ± 0.51^NS^
Cholesterol (mg/dL)	76.9±4.50^∗∗∗∗^	77.5±5.14^∗∗∗∗^	64.5±3.75^∗∗∗∗^	135.0 ± 7.19	92.5±5.86^∗∗∗^
Triglycerides (mg/dL)	82.5±4.21^∗∗∗∗^	89.1±3.50^∗∗∗^	79.5±5.20^∗∗∗∗^	122 ± 8.25	90.7±6.16^∗∗∗^

The significance of difference was analyzed by one-way ANOVA and Dunnett test (compare all vs. Ehrlich group). Values are expressed as means ± SEM. *N* = 6 observation for each group. One-way ANOVA was significant at *P* < 0.05. Dunnett test was significant from the corresponding Ehrlich group value at ^∗^*P* < 0.05, ^∗∗^*P* < 0.01, ^∗∗∗^*P* < 0.001, and ^∗∗∗∗^*P* < 0.0001.

## Data Availability

The data used to support the findings of this study are available from the corresponding author upon request.
